# “Life-changing”: the experience of super-responders to biologics in severe asthma

**DOI:** 10.1186/s12890-022-02241-2

**Published:** 2022-11-28

**Authors:** Joseph W. Lanario, Lucy Cartwright, Rupert C. Jones, Ross Sayers, Michael E. Hyland, Matthew Masoli

**Affiliations:** 1 Faculty of Health, University of Plymouth, Room N10, ITTC Building, Plymouth Science Park, Plymouth, Devon, PL6 8BX UK; 2 Plymouth Marjon University, Plymouth, UK; 3 Department of Respiratory Medicine, Royal Devon and Exeter Hospital, University of Exeter, Exeter, UK; 4 Royal Devon and Exeter Hospital, University of Exeter, Exeter, UK

**Keywords:** Severe asthma, QoL, Patient perspectives, Qualitative, Biologics, Super-responders

## Abstract

**Background:**

There is limited information on the patient’s perspective of how biologic treatments impact their lives. We conducted a qualitative study to explore the patient’s experience of being considered a super-responder from a quality of life perspective.

**Methods:**

Patients with severe asthma identified as super-responders were invited to semi-structured interviews conducted online. Participants could bring a family member/friend to the interview. The interviews explored experiences of biologic treatment, were transcribed and underwent thematic analysis.

**Results:**

Twenty-five participants took part in this study. Themes emerged on the impact of biologic treatment for participants and for their friends/family: (i) Words used to describe their often life-changing experiences and (ii) the positive changes noted. Biologic treatment stopped the disruption of family life and social life caused by exacerbations. Improvements in mental health were also noted. Marked individual variations in the way it affected their lives were noted. Most participants noticed improvements 2–3 months after starting their biologic, but some noticed improvement within a few days and others after 6 months.

**Conclusions:**

Super-responders reported profound but heterogeneous improvements following biologic treatment beyond asthma symptoms and exacerbations including important benefits to social and family life. Improvements may be underestimated as social and family benefits are not reliably measured in current studies with implications for health economic evaluations. Not all patients are super-responders, and excellent responses may be lost in group mean data in trials. Individual time course and response patterns need further elucidation to identify who will respond best to biologics.

**Supplementary Information:**

The online version contains supplementary material available at 10.1186/s12890-022-02241-2.

## Background

People with severe asthma constitute 5–10% of the global asthma population, approximately 10–14 million people worldwide [1]. A diagnosis of severe asthma is made when a patient is on high doses of conventional treatment, their symptoms persist and other explanations for these symptoms have been ruled out [1]. Biologic treatments are now available to treat these people, but clinicians have observed that response ranges from no response to profound improvements in health.

Biologic treatments significantly decrease exacerbations, hospitalisations, systemic steroid burden, and non-respiratory symptom burden [2–4]. Despite these observations, group mean data from randomised controlled trials (RCTs) show that quality of life benefit is variable and often fails to meet the minimum clinically important difference (MCID) for improvement compared to placebo [5]. Severe asthma is a heterogeneous disease and response to biologic treatment varies between patients [6]. There is emerging real-world evidence that quality of life does increase after biologic initiation for a significant number of patients [7].

So far, efforts to define the super-responder population have mainly used traditional objective clinical outcomes. A consensus paper on the management of severe eosinophilic asthma recommends a traffic light system for patients that suggests assessment 4 months after starting a biologic treatment [8]. Three categories and courses of action are suggested: (i) Non-responders, for whom therapy should be discontinued; (ii) Intermediate responders for whom therapy should be continued for a year and then reassessed; (iii) Super-responders for whom therapy should continue. The categories are not defined, but lung function, symptoms, quality of life and exacerbation history are suggested as metrics for judging response. These recommendations are in line with the ERS/ATS guidelines on the management of severe asthma which proposes that similar outcome measures are used in clinical trials [9].

A recent survey of clinicians conducted as a Delphi process, concluded that criteria for a super-responder should include eliminating all asthma exacerbations, large improvement in asthma control (≥ 2xMCID) and cessation of oral steroids [10]. Other suggested criteria include 75% reduction in exacerbations, having well-controlled asthma, and 500 ml or greater improvement in FEV1. The Delphi process did not come to a consensus on whether quality of life should be included as an outcome. In contrast, a survey of patients found that improving quality of life was the most important outcome to people with severe asthma. Improving quality of life was ranked higher than decreasing the number/severity of asthma exacerbations and being able to participate in physical activity [11].

Qualitative studies have captured the burdens of severe asthma from a patient perspective including: daily symptoms, frequent attacks, hospitalisations, oral corticosteroid (OCS) burden and disruption to family life [12, 13]. While recent qualitative research has explored patient perspectives on the benefits of a range of different add-on pharmacotherapies [14], no studies have specifically examined the response to biologics. In this study, we used qualitative methods to explore the experiences of a population of super-responders to severe asthma biologics.

This study had three aims in relation to the way individuals responded to biologic treatment:

1. To explore what it is to be a super-responder from a patient’s perspective.
2. To describe the time course and nature of improvements including respiratory and non-respiratory symptoms.
3. To explore the experience of family members since their relative started a biologic treatment.

## Methods

### Study design

This qualitative study used semi-structured interviews to explore the experience of patients deemed super-responders to their biologic treatments. Interviews were conducted using Zoom video call software [15].

### Recruitment and selection

All participants were aged ≥ 18 years, under the care of a specialist severe asthma service and receiving a biologic for severe asthma. Patients were discussed in a multi-disciplinary team (MDT) meeting to determine if they were considered a super-responder and eligible to take part in the study. Criteria for making this judgement included all the following: significant improvements in quality of life (as measured by the Severe Asthma Questionnaire (SAQ) [16] (≥ 2xMCID of 0.5) [17], an improvement on the Global rating of change (GRoC) scale of a ‘good’ or ‘great’ deal better [17] and > 50% reduction in OCS usage. If eligible to take part, patients were provided with information about the study and told that a significant other person (e.g., partner) was invited to take part simultaneously. Information was provided for the partner. Patients and significant others who provided informed consent were enrolled into the study. Informed consent was provided by both parties for anonymised quotes to be used for research. Participants unfamiliar with video call software were first invited to a test call to troubleshoot any technical problems.

Recruitment took place between 6th July and 10th September 2021.

### Data collection

Quantitative data were routinely collected from participants receiving biologic treatment at the recruiting hospital. These data are reported to describe the participants and their response to biologic treatment.

The role of conducting the interviews was split evenly between two researchers (MH and JL) with the other person listening in to the interviews. Our semi-structured interview guide was discussed and refined by seven patients with severe asthma. The version used in this study can be found in the Additional file 1. When present, a family member or friend was invited to comment unless they spontaneously provided comments. Participants were asked to provide information about anything they felt relevant without being asked a question by the interviewer. Apart from the specific questions, participants were encouraged to talk by the interviewers using linguistic and paralinguistic cues showing interest. Neither MH nor JL had an established relationship with any of the participants prior to study commencement. The interviews lasted between 20 and 60 min and were audio-recorded and transcribed verbatim. The transcripts were anonymised before analysis.

### Analysis

Content analysis was used, with transcripts undergoing thematic analysis [18] by two researchers (JL, LC). NVivo version 12 was used to code the interviews and help organise themes and illustrative quotes. Recurring themes and illustrative quotes were added to the NVivo file by one researcher, before being discussed and agreed with the other. JL and LC had previously received training in how to use NVivo. All the authors familiarised themselves with the transcripts by reading them multiple times and discussed and approved the final themes.

### Ethical approval

This study received ethical approval the Research Ethics Committee/Health Research Authority (REC reference: 21/WA/0147, IRAS ID: 280843) and was sponsored by the University of Plymouth.

## Results

Forty five patients were identified and invited; the first 25 to respond were recruited. Fourteen participants had a family member or friend join them for the interview. Table 1 shows the characteristics of the patient sample including percentages and range where appropriate. Table 2 shows mean scores of clinical variables outcome measures before and after treatment with standard deviations (SDs) and range where appropriate.

**Table 1:** Demographics and co-morbidities (N = 25)

		n (%)
Biologic	Omalizumab	2 (8)
	Mepolizumab	7 (28)
	Benralizumab	15 (60)
	Dupilumab	1 (4)
Gender and age	Male	9 (36)
	Age 18–34	1
	35–49	1
	50–64	3
	65–79	4
	Female	16 (64)
	Age 18–34	2
	35–49	3
	50–64	7
	65–79	4
Ethnicity	Caucasian	24 (96)
	Mixed race	1 (4)
Age at onset	Adult	17 (68)
	Child	8 (32)
Smoking	Never	17 (68)
	Ex	8 (32)
	Current	0
Nasal polyps		Present 7 (28)
		Absent 18 (72)
		Mean (range)
Age		56 (56–77)
BMI (Kg/m^2^)		29.6 (21–47)
Maximum Eosinophils in 12 months preceding biologic		0.78 (0.2–1.48)
Months since initiation^#^		22 (7–44)

^#^4 participants had previously been on at least one other biologic

**Table 2:** Pre and post-biologic scores on outcome measures, means and (range), (SD)

	Pre biologic	Post biologic
	n (%)	
Participants requiring maintenance OCS	12 (48)	5 (20)
	Mean (range)	
Maintenance OCS dose (mg/d)	12.5 (5–30)	5 (0–10)
Exacerbations requiring OCS in last 12 months	8 (4–16)	0.3 (0–1)
	Mean (SD)	
FEV1-Absolute (L)	2.37 (0.70)	2.47 (0.77)
% Predicted	79.4 (18.80)	81.2(25.15)
ACQ 6 (0–6)	2.60 (1.15)	0.81 (0.83)
SAQ (0–7)	3.81 (1.26)	6.01 (0.91)
SAQ Global (0–100)	53 (21.38)	88 (12.59)

The MCID for the ACQ and SAQ is 0.5 and for the SAQ-global is 11

The impacts of severe asthma before participants started a biologic are presented below. Four themes are presented, two concerning the experience of the patient participants (Tables 3 and 4), and two on the direct observations made by their family/friends (Tables 5 and 6). We provide evidence for the time course and nature of reported improvements in participants’ health.

**Table 3:** Theme 1: unprompted words used to describe their biologic treatments and/or its impacts

“Life changing”, “changed my life”	“I, I just said the first time I went back to the um, Dr [Name] I said, “It’s, it’s life changing.”
	“I’ve been completely blown away with how much this drug has changed my life” [PPT2]
	“And, and then by the second one I, second injection I thought, well, do you know what, this is really changing me now like, my life, my life is seriously changing” [PPT4]
	“I’m so grateful to be able to contribute to this. It has changed my life, in has in a way changed your life but for me it’s huge. It’s just huge. It’s such a relief.”[PPT8]
“Miracle cure” or “close to cure” or “wonder drug”	“I mean seems like a bit of a miracle drug you know. It’s extraordinary.” [PPT22]
	In reference to speaking to hospital staff since taking the injection: “Now when I go up they say ‘are you alright because we haven’t seen you?’ (laughter) I said, “you’re not going to, I’ve got this miracle cure and you’re not having it back” (laughter)[PPT1]
	“But for me, this is as close to it curing asthma as I think we’ll probably ever get.”[PPT7]
	“I want to go and worship at some god who created this. (laughter)” [PPT 19]
	“I can’t say no more. I think it’s just brilliant. That’s why I call it my wonder drug. That’s all on my consultant notes. My wonder drug.”[PPT11]
	“I can’t praise Mepo enough to be honest it’s, it’s such a, it’s a wonder drug really.”[PPT6]
	“It’s a wonder drug really”[PPT23]
“Amazing”, “mind blowing”	“Amazing, amazing, really well, yeah, I am”[PPT18]
	“Erm, yeah, it’s just been totally mind blowing actually” [PPT5]
“Life back”, “second life”	“I have my life back” [PPT19]
	“It’s er, it’s sort of given me a sort of a second, second life in old age really”[PPT16]
“Asthma stable”, “better”	“my condition’s stabilised I think, yeah.” [PPT20]
	“I would say I’m better. If that makes sense” [PPT13]

**Table 4:** Theme 2: positive changes since commencing biologic treatment

*Physical health improvements*	
Reduction in limitations from daily asthma symptoms	“It’s wonderful to be able to get up and run out to the garden. I can do so much more without the breathlessness” [PPT1]
Other asthma treatment not needed	“I was getting through the inhalers like there was no tomorrow. Now I’ve got one in the cupboard and I’m having to look at them to make sure they’ve not gone out of date because I’ve not used it. It’s amazing.” [PPT3]
Fewer/less severe colds/chest infections	“I haven’t had to go back to the GP for any chest infections since I, at all, since I’ve been on the injection.” [PPT16]
Able to be more physically active	“I could never have done the coastal path, but I was able to do that within three weeks of the injection.” [PPT12]
Decreased exacerbations and hospitalisations	“I would have probably been in hospital at least once by this time, certainly had the ambulance here a couple of times and I would have been on courses of Prednisolone by now. None of it, none of the above.” [PPT19]
Improvement in other health conditions	“One of the most positive side-effects for me is that also suffer from eczema and since starting the injections it’s completely cleared up”. [PPT17]
*Mental health improvements*	
Feeling more confident	“It’s just given me this level of confidence that I don’t have to consider asthma in anything that I undertake.” [PPT4]
Less worry, anxiety and fear	“So Mepolizumab has largely, really not just given my life back in terms of respiratory function but also the anxiety, it’s really helped with that.” [PPT22]
Feeling happier	“And happier obviously because I’m not taking inhalers all the time.” [PPT8]
Less embarrassment	“It was really embarrassing, that’s the psychological aspect of it … my colleagues all used to say ‘your cough, your cough is awful’ … that’s one aspect that doesn’t happen. It’s marvellous, wonderful.” [PPT3]
Empowerment	“Asthma was my controller. Now I’m the controller.” [PPT4]
*Social improvements*	
Able to socialise more	“Before, being on steroids and being quite depressed, a lot of the time I would pull out of social things… whereas now I’m just saying yes and I can do everything and I can go out and I can have fun and I can see my friends more.” [PPT2]
*Family improvements*	
Less worry for family	“The rest of the family are more relaxed now.” [PPT10]
Able to care for family	“With my chest feeling better I was then able to better care for him. It felt good to be able to do that instead of having to rely on him to look after me.” [PPT1]
*Improvements in working life*	
Able to be more productive in work	“Now I have this attitude of there’s no limits and I mean my boss loves it because he says ‘can you do this?’ and I’m ‘yeah, sure, why not’.” [PPT2]
Able to stop working	“I just thought ‘this is my one shot to enjoy some retirement’ … by four months I realised that I didn’t know what the longevity of the effects of the injection was gonna be. I decided to retire to maximise it.” [PPT4]

**Table 5:** Theme 3: words used by family/friends to describe the impact of biologic treatments and/or its impacts on the participant

Life-changing	“I’d personally say looking from the outside I’d describe it as life changing, significantly. There’s no doubt about it.” [S14]
Asthma has been ‘switched off’	“It is like you’ve literally switched the asthma off.” [S5]
Patient is like a different person	“After the injections I just thought as time went on, this is a completely different human being.” [S14]
Patient is like they were before	“…literally was doing nothing without being under huge pressure for coughing … to taking this injection and it completely put her back to the woman she was before all this started.” [S12]
Patient’s quality of life is better	“It gives you quality of life … whereas before she lived clearly but it was always with this threat.” [S19]

**Table 6:** Theme 4: views of family on improvements noted in the participants

*Physical health improvements for patient*	
Reduction in daily asthma symptoms	“The most significant thing you have in life is to be able to breathe, to be able to walk around and unable to breathe for how many years, fifty two years?” [S14]
Decreased exacerbations and hospitalisations	“There haven’t been any hospital trips whereas before it was quite regular, every two or three months she’d be in hospital, and I’d be looking after the kids and trying to work and that.” [S8]
Able to be more physically active	“We do something which is run-walking, so you do a run and space it out with a walk and run. I would say before when she was on medication, she wouldn’t be able to do it, whereas now I can’t keep up with her.” [S23]
Fewer/less severe colds/chest infections	“If you have anything it might be a cough … nose-blowing but nothing like you used to.” [S12]
*Mental health improvements for patient*	
Feeling more confident	“I’ve not noticed you worry that if you’re a little bit off that would turn into something more massive. I think you’re more confident that you can deal with it now, rather than before you were quite worried about these things.” [S9]
Feeling happier	“I think generally now we see a much happier person.” [S2]
Less worried	“You don’t have to worry about it so therefore you’re more relaxed.” [S17]
*Impact for family member themselves*	
Less worry/stress for family	“She’d worry because if she was poorly and the kids were at school and needed to be picked up I’d have to come away from work and then I’d be worried that I’d have to make up the time and she’d be worried that I was worried about that. It’s made a big difference.” [S8]

## Before commencing a biologic

Participants described the impact severe asthma had on their lives before starting a biologic treatment, in particular breathlessness and disturbed sleep.

“I was having problems with the breathing, very breathless and this was at the beginning of last year before the first injection” [PPT15]

“I was sleeping so badly at night with the wheezing I wouldn’t get up until lunchtime because why get up when I couldn’t do anything?” [PPT 19]

It was common for participants to report symptoms that limited their mobility and physical activity:

“I mean if I was poorly I wouldn’t be able to get up my stairs, I would take, I would have to stop to go up my stairs.” [PPT 18]

“…it got to the point where you know, if I was er, planning to go into town or anything like that with the wife, erm, I’d get sort of 200 yards up the road and er, I would have to give up” [PPT16]

Some participants described becoming habituated to their level of morbidity and accepted it as part of daily life:

“I phoned up my son he was so used to ‘dad, you do sound so out of breath and wheezy’ I say I suppose I am, but it’s been going for so many years I take that as a norm I’m a bit tight.” [PPT10]

Frequent chest infections and/or colds were also problematic:

“And a regular occurrence would be chest infections from October onwards. There would be a lot of steroids and antibiotics” [PPT11]

In combination with the daily symptoms, frequent asthma exacerbations and colds caused disruption to participants’ working lives:

“I think that probably the impact that it had on my job, my asthma before and after, so I had hugely low attendance levels.” [PPT8]

Due to the severity of symptoms, participants described feeling unable to socialise out of fear of encountering asthma triggers. An example one participant gave was avoidance of their friend’s dog:

“So yeah, had to, had to stop going to their house.” [PPT17]

Participants also described struggling to engage in many aspects of family life:

“…I felt like a burden especially when (Name, Participants Partners Mum) had to come and stay because I couldn’t even get to the bathroom, she was looking after the kids.” [PPT8]

Family members were also aware of their loved ones struggles with asthma, with one grandchild explaining they did not know where their Grandmother lived as she was so frequently in hospital:

“I was never out of hospital, me and my grandson were talking about it last night and he said, ‘actually Gran I didn’t know where you lived’ (laughter)”

Fear of death from an asthma attack was frequently reported:

“I was struggling, I remember um, lying in a shower room gasping for breath. Thinking, I’m going to die here.” [PPT 6]

Children were also aware of the severity of their parents’ asthma attacks and had concerns for their parents’ wellbeing:

“I was in hospital with everybody listening and she said, mummy you’re not gonna die, are you?” [PPT5]

The burdens of asthma described by these participants were all-encompassing, profoundly impacting the individual and their family.

## The experiences of super-responders

Theme 1: Words used to describe their biologic treatments and/or its impacts.

Table 3 shows the words and phrases used by participants when reflecting on their response to biologic treatments.

Theme 1 conveys the dramatic benefit the super-responder participants experienced after starting their biologic treatment. Participants used strong words and phrases to express this impact, some of which bordered on religious terminology, e.g. “miracle cure”. Of the words and phrases used, “life-changing” was the most commonly used, followed by “miracle cure”, “close to cure” and “wonder drug”.

However, some participants were more reserved in how they expressed the impact of the biologic, stating that their asthma is now “stable” or that they were now “better”.

Theme 2: The range of positive changes since commencing a biologic treatment.

Participants described improvements in different aspects of their lives following biologic treatment. These included physical and mental health improvements, as well as improvements to their social, family and working lives, summarised in Table 4.

Participants described reductions in their daily symptoms and the frequency/severity of colds and chest injections after staring a biologic treatment. In terms of mental health improvements, participants reported increased confidence and decreased fear/anxiety, which they attributed to their decreased asthma symptoms.

For many participants, the realisation that their health had improved was an emotional experience:

“…I have a flattish garden on a slope and then it goes quite steeply up hill. I’ve never walked up there without a terrible effort, I could just stride up and I cried.” [PPT 19]

When describing improvements to their physical and mental health, participants framed this improvement in the context of how their health was before commencing their biologic treatment (see bolded text):

Because I can breathe so well I obviously now I can walk up hills which I couldn’t do before.” [PPT3]

My asthma doesn’t really cross my mind like it used to … that has improved, because I don’t have that constant worry that I used to have with it.” [PPT7]

These improvements were described alongside reduced use of asthma medicines, particularly inhalers and increased engagement in social and family activities:

“It’s been absolutely life changing, totally, and for my family, that would have had to look after the children, look after the dogs, the whole, you know, visit, or their visiting and all their babysitting and things.” [PPT18]

Some participants expressed having more energy and attributed this to their improved sleep quality due to decreased asthma symptoms at night:

“I’ve got more energy now cause I’m sort of sleeping better cause I haven’t got the hacking cough” [PPT5]

## Time course of benefit

Most participants reported that they noticed improvement 2–3 months after starting a biologic treatment:

“…it was probably at the end of month two, maybe beginning of month three” [PPT17]

However, some participants reported experiencing benefit a matter of days after their first injection:

“…my manager who is also a friend said ‘your voice sounds different’ so even the way I talked and the tightness of my breath was noticed within twenty four hours.” [PPT8]

“Anyway, the first injection went in and two days later the wheezing stopped”. [PPT19]

Whereas others did not notice improvement until 6 months after their first dose:

“And I’d say almost, possibly after six months, I did start to notice a little bit that I wasn’t being admitted so many times to hospital and the admittances were starting to get further and further apart.” [PPT1]

Some participants contrasted the fast reduction in their asthma symptoms to the greater time it took for their mental health to improve:

“I would say maybe a year, maybe eight months to a year that kind of thing. It didn’t happen overnight at all. The respiratory happened over say four months or so but the anxiety has taken a while actually to you know.” [PPT22]

It took time for many participants to realise how much their health had improved and to regain confidence:

“I started to slowly realise that I could go into the sea and I wouldn’t drown, and I could go for a walk on my own and I wouldn’t collapse and you know, so very, very slowly the change in my life certainly um, was the lack of anxiety.” [PPT12]

## Observations made by family and friends

Theme 3: Words used by family/friends to describe their biologic treatments and/or its impacts. This is summarised in Table 5.

Life-changing was the most common phrase used by patient participants to describe the changes they have experienced since commencing the biologic. This phrase was also used by and family/friends.

Friends and family members commented on the improvements they had seen in the participants. See Table 6.

As with the patient participants, family and friends noticed improvements in the patient participant’s physical and mental health. Family members reflected on how their partner’s improved health also improved family dynamics by making childcare easier. Patient participants’ confidence in managing their own health was also observed by friends/family to have improved. Unlike the patient participants, family members did not raise the impact their partner’s severe asthma, or its treatment, had on their social life.

## Discussion

In this study, super-responders provided narratives on the range and time course of their response to biologic treatments. There is a lack of research in to patient perceptions of response to biologic treatments for severe asthma [19]. While previous qualitative research has collected data from patients on various add-on therapies [14, 20], this is the first study to focus on super-responders. The phrase “life-changing” and alternatives were frequently used by participants and their family/friends. This experience was characterised by reductions in asthma symptoms, chest infections, acute exacerbations and improved mental health. In many cases, participants described the elimination of daily symptoms, chest infections and exacerbations entirely. The participants in this study demonstrate that asthma remission [21] is a feasible treatment goal.

Compared to a recent study investigating predictors of response to Anti-IL5 drugs, participants in this present study had higher peak eosinophils in the previous 12 months and a higher exacerbation frequency [7]. Despite this, the participants in the present study described profound improvements to their quality of life following biologic treatment.

When describing the benefits of biologic treatments, participants focused on the physical improvements to their health. Most of the participants we interviewed described feeling benefit from their biologic after 2–3 months. Others reported benefits within as little as 2 days, or as long as 6 months, indicating that early response is not always the precursor of good response. This demonstrates that it may be possible to identify super-responders within 4 months of commencing a biologic treatment. Further quantitative research is needed to investigate the time course to improvements and identify for whom and when good response may be predicted.

Improvements to participants’ mental health took longer than improvements to their physical health. The persistence of anxiety despite the elimination of asthma symptoms is understandable and has also been observed in patients following cancer remission [22]. This finding, as well as the finding that there is great variability in the time it took to respond to a biologic, have implications for patient education before commencing a biologic.

Severe asthma can limit a person’s ability to engage in family life [13], including taking on childcare responsibilities [23], and concerns that they are a burden [24]. Participants in this present study described similar problems before commencing their biologic, but also how they reconnected with family once their symptoms decreased. This was noticed and appreciated by partners. The burden on family life is not assessed in clinical trials, which means the benefits of biologics on this aspect of patient’s lives is unappreciated. The impact of witnessing life-threatening attacks on their families, especially young children, were profound. Childhood stress is associated with poorer health in later life [25] and it is possible that witnessing a parent have asthma attacks leads to long term health consequences for a child. Further research is needed to investigate this and how improvement in a parent’s asthma benefits the family.

The range of benefits on quality of life reported were as wide as the range of different lives participants led and the aspirations they held. For one person it was to walk the coastal path, go surfing, or have a normal social life, for another to just be able to walk across a room without breathlessness. Participants were able to resume physical activity following biologic treatment and this was the second most important treatment outcome for people with severe asthma [11]. Exercise was often a social activity undertaken with friends or their partner, or while playing with their children. Participants thought of treatment success as being able to do the activities that mattered to them, rather than in terms of objective clinical improvement [26].

With respect to quantitative improvement there was an average increase in FEV1 of 100 ML on biologics from a baseline of 2.37L (79.4% predicted). This modest improvement does not reflect the profound improvement experienced by participants and highlights the discordance between lung function and quality of life. In contrast, two scores of quality of life (SAQ and SAQ-global) increased by 4 × and 3 × their respective MCID values, and better captured the experiences of participants.

The impact of biologics on patients’ lives merits further research. However, there is discordance between what is most important to people living with severe asthma and what is prioritised by clinicians [11, 26]. This is apparent from a clinician-led definition of super-responders [10]. If super-responders are identified using only objective clinical outcomes, data from this study suggests that many of the benefits of biologics treatments will be missed. Criteria for identifying super-responders must include Health-Related Quality of Life (HRQoL) if the wider benefits of biologics are to be captured. This also has implications for health economic evaluations which are often based on QALYs gained from a treatment.

Without a consensus definition, it is difficult to estimate the number of super-responders present in RCTs. Group mean data from trials may hide the participants who have life-transforming benefits. However, once a consensus definition is agreed, super-responders can be identified and sub-analyses of their data can be conducted. This will facilitate further research into predicting who will respond to which biologic. With the advent of personalised medicine [27] it becomes increasingly important to accurately measure the wider HRQoL benefit so that expensive resources are targeted to those with most to gain. It is clear that people with severe asthma and clinicians have different priorities for treatment goals yet at present the patient voice is not represented in discussions about them.

### Limitations

The participants in this study were from a single centre and predominantly Caucasian. Further research is needed to determine if the results are consistent across different ethnicities and cultures. Due to the lack of consensus on the definition of a ‘super-responder’, participants were selected on the basis of an MDT decision after reviewing their patient-reported outcomes. Lastly, due to the time period that the interviews were conducted, some participants had been shielding during the Covid-19 pandemic. As such, it was difficult for them to say whether they had not developed a seasonal chest infection due to the biologics or because they were social distancing. Conversely, it was also not possible for some participants to take full advantage of their improved health due to shielding and anxiety related to COVID.

## Conclusions

While previous definitions of super-responders have been clinician led [10], this is the first study in which people with severe asthma have described the experience in their own words. This study demonstrates the life-changing effects biologics can have on people’s lives. The important improvements for patients go beyond respiratory symptoms; the impact on their working, social and family life is profound. The patient voice should be heeded in the assessment of response to biologics if these benefits are to be captured.

The nature and time course of response show marked heterogeneity. Major benefits in some individuals may be lost in group mean data from trials and sub-analyses of super-responder data is needed to explore this group’s response to biologics. Larger studies of super-responders are essential to elucidate characteristics which may be used to identify and predict response.

## Supplementary Information

**Additional file1**. Semi-structured interview guide.

## Data Availability

The data set can be requested from the corresponding author.

## Abbreviations

RCT: Randomised controlled trial
ERS: European Respiratory Society
ATS: American Thoracic Society
FEV1: Forced expiratory volume in 1 second
MCID: Minimum clinically important difference
OCS: Oral corticosteroids
MDT: Multi-disciplinary team
SD: Standard deviation
SAQ: Severe Asthma Questionnaire
ACQ: Asthma Control Questionnaire
GRoC: Global rating of change
REC: Research Ethics Committee
IRAS: Integrated Research application System
BMI: Body mass index
