# Supplementary material for: “Life-changing”: the experience of super-responders to biologics in severe asthma
Source: BMC Pulm Med. 2022 Nov 28;22:445. doi: 10.1186/s12890-022-02241-2 (PMC9702657; doi:10.1186/s12890-022-02241-2)
Supplement: Supplementary file 1 — Additional file1. Semi-structured interview guide. [file 12890_2022_2241_MOESM1_ESM.docx]

**Supplementary Material: semi-structured interview guide**

**Super-responder questions – order to be adjusted to the conversation with the participant**

When injections started?

What to expect?

When did they first notice a difference?

- Asthma
- Mood
- Key event

Probe with partner if present.

What was the difference?

Did it happen gradually? Was there improvement over the next few months or did it peak early on?

Any side effects from taking the injection next day etc.?

Experience with frequency and severity of colds?

Any change in any other symptom not necessarily related to asthma?

Experience with allergies

Any reduction in effectiveness just before the next injection?

Anything you would want other patients to know about the injections?

One word describing how they are different now from what they were before

Probe this question with the partner

Anything else?

Note: let them talk about anything however irrelevant.

Thanks and how helpful they have been
